# Therapeutic efficacy of ^166^Holmium siloxane in microbrachytherapy of induced glioblastoma in minipig tumor model

**DOI:** 10.3389/fonc.2022.923679

**Published:** 2022-11-07

**Authors:** Mehrdad Khoshnevis, Richard Brown, Sara Belluco, Ilyes Zahi, Luca Maciocco, Catherine Bonnefont-Rebeix, Elodie Pillet-Michelland, Jonathan Tranel, Thierry Roger, Christophe Nennig, Patrick Oudoire, Lionel Marcon, Olivier Tillement, Cédric Louis, Hélène Gehan, Manuel Bardiès, Maurizio Mariani, Valeria Muzio, Jean-Philippe Meunier, Charlotte Duchemin, Nathalie Michel, Estelle N’Tsiba, Ferid Haddad, Thierry Buronfosse, Claude Carozzo, Frédérique Ponce

**Affiliations:** ^1^ Université de Lyon, VetAgro Sup, UR ICE, Marcy L'Etoile, France; ^2^ Inserm, UMR1037 CRCT, Toulouse, France; ^3^ Advanced Accelerator Applications, a Novartis Company, Saint-Genis-Pouilly, France; ^4^ EVEON, 305 rue Aristide Berges, Montbonnot Saint Martin, France; ^5^ Institut Lumière Matière, UMR CNRS 5306, UCBL, Campus LyonTech - La Doua, Villeurbanne, France; ^6^ Nano-H SAS, 305 rue des Fours, Fontaines Saint Martin, France; ^7^ Advanced Accelerator Applications, a Novartis Company, Colleretto Giacosa, France; ^8^ Subatech, CNRS/IN2P3, IMT Atlantique, Université de Nantes, Nantes Cedex, France; ^9^ IP Arronax, Saint Herblain, France; ^10^ Université de Lyon, VetAgro Sup, Laboratoire de Biologie Médicale, Marcy L'Etoile, France; ^11^ Université de Lyon, VetAgro Sup, Service de Cancérologie, Marcy L'Etoile, France

**Keywords:** glioblastoma, U87 cells, minipig, 166Ho, microbrachytherapy, animal model

## Abstract

Glioblastoma is considered the most common malignant primary tumor of central nervous system. In spite of the current standard and multimodal treatment, the prognosis of glioblastoma is poor. For this reason, new therapeutic approaches need to be developed to improve the survival time of the glioblastoma patient. In this study, we performed a preclinical experiment to evaluate therapeutic efficacy of ^166^Ho microparticle suspension administered by microbrachytherapy on a minipig glioblastoma model. Twelve minipigs were divided in 3 groups. Minipigs had injections into the tumor, containing microparticle suspensions of either ^166^Ho (group 1; n = 6) or ^165^Ho (group 2; n = 3) and control group (group 3; *n* = 3). The survival time from treatment to euthanasia was 66 days with a good state of health of all minipigs in group 1. The median survival time from treatment to tumor related death were 8.6 and 7.3 days in groups 2 and control, respectively. Statistically, the prolonged life of group 1 was significantly different from the two other groups (p < 0.01), and no significant difference was observed between group 2 and control (p=0.09). Our trial on the therapeutic effect of the ^166^Ho microparticle demonstrated an excellent efficacy in tumor control. The histological and immunohistochemical analysis showed that the efficacy was related to a severe ^166^Ho induced necrosis combined with an immune response due to the presence of the radioactive microparticles inside the tumors. The absence of reflux following the injections confirms the safety of the injection device.

## Introduction

Glioblastoma (GB) is the most common primary brain tumor and one of the most resistant cancer of the central nervous system to standard treatments (1–3). It accounts for 45% of malignant primary central nervous system (CNS) tumors, and 54% of all gliomas. The average incidence rate of GB is 3.2 per 100,000 with an estimation of 12,000 new cases per year (4). Unfortunately, GB is associated with extremely poor prognosis, and long-time survivals after diagnosis are rare (5). The gold standard treatment of GB including surgery, radiotherapy and chemotherapy increased the median overall survival to 14 months (1, 6–8). However, current treatment remains palliative and patients often relapse after a few months. Recently, new treatments have emerged, such as immunotherapy. However, results for these new modalities are not yet conclusive.

In the last decade, radiotherapy has been considered the most effective adjuvant treatment to increase the survival rate of malignant glioma patients (9). In the 1970s, the absorbed dose-effect relationship in GB radiotherapy was investigated (10, 11). A target absorbed dose of 60 Gy into the tumor was associated with increased survival and evaluated as the optimal radiation dose. This increased life expectancy was not associated with significantly increased toxicity. In general, according to different publications, absorbed dose escalation up to 100 Gy resulted in increased toxicity without any survival benefit (12, 13).

Unfortunately, in most external beam radiotherapy (EBRT), side effects such as cerebral edema occur due to the irradiation of normal tissue around the tumor, resulting in tissue necrosis (14–16). In this regard, brachytherapy (BT) and other targeted approaches are now considered for the treatment of cerebral tumors to prevent collateral damage. However, for BT a complicated surgery is required to insert the radioactive element inside the tumor, potentially resulting in side effects and even worsening of neurological signs. An alternative solution to overcome this issue would be the use of liquid radioactive sources using a novel method, so-called microbrachytherapy (mBT). This method consists in injecting radioactive microspheres or microparticles suspended in liquid, instead of the radioactive grains typically used in BT. In this context, ^166^Holmium (^166^Ho) is an attractive radioelement. In our previous studies (17, 18) we explained the ^166^Ho characteristics that make it a suitable radioactive source for mBT.

This study’s first objective was to demonstrate that mBT using ^166^Ho is safe and effective for treating induced GB in Yucatan minipigs, as a preclinical study prior to attempting clinical trials on patients with GB. GB model was induced by implantation of human U87 cells line that is considered as one of the most radioresistant cell lines (19–21).

## Materials and methods

### Animals

This study was carried out in twelve Yucatan minipig (INRA Saint-Gilles, France), both male (n=7) and female (n=5), as a GB model. Experiments were performed in a preclinical platform (Claude Bourgelat Institute in Marcy l’Etoile, France) and conducted in accordance with the French Ministry of Scientific Research (2015052012034148 v1) and approved by the VetAgro Sup Ethical Committee (1522 V2). All aspects of the maintenance and use of the animals, including surgical procedures and pain assessment were performed and monitored in compliance with French regulations (transposition of Directive 2010/63/EU) and the local Animal Welfare Body. The animal care and welfare condition during all the experimental studies were the same as previous studies (17, 19).

### U87 cells culture

U87 cells were obtained from the American Type Culture Collection (U-87 MG, ATCC^®^ HTB-14™, LGC Standards, Molsheim, France) and were prepared as previously described to induce glioblastoma on Landrace pigs and Yucatan minipigs (17, 19). Briefly, 35–50 × 10^6^ U87 cells were harvested from two T175 tissue culture flasks after one week in growing medium (Minimum Essential Media (MEM) supplemented with 10% fetal calf serum, 100 U/ml penicillin, 100 μg/ml streptomycin, 2 mM l-Glutamine and 0.25% g/mL amphotericin B) with three medium changes. Cells were processed with 0.25% trypsin-EDTA to be harvested and then washed twice in PBS before being pelleted for 4 min at 3000 g in a 2 ml Eppendorf vial just before the inoculations. The final cell concentration in each Eppendorf vial was about 1.7 × 10^6^ cells/10 μl. For each subject, 40 μl of the cells were injected into the right hemisphere.

### ^165^Holmium microparticles suspension preparation

Before the activation process that produces radioactive ^166^Ho, the ^165^Holmium (^165^Ho) microparticles suspension as a stable isotope of Ho was prepared as previously described (17, 18). Briefly, nanostructured Ho2O3 precursor (0.397 mol) was slurried and refluxed in 1.5 L ethanol together with acetic acid (0.262 mol) and Si-EDTA (0.05 mol). It was allowed to cool down to ambient temperature and then transferred into 200 mL plastic centrifuge bottles. Particles were washed in ethanol twice by centrifugation cycles of 10 minutes at 4,100 rpm and then resuspended in 160 mL mQ water. Particle size was homogenized by stirring the suspension for 4 days at 30°C. The objective was to synthesize particles with high ^165^Ho content. The particle mean size was 470 nm. The dry matter concentration (i.e., the solid phase once completely dried) was 550 ± 50 g/L consisting of 28 ± 3% Ho weight content. This elevated fractional weight of Ho enables the delivery of a highly activated ^166^Ho dose to tumor cells within a low and suitable volume unit of treatment. Density of the final ^165^Ho microparticle suspension was 1.38 g/ml, measured in triplicate at 25 °C by weighing 1 ml of suspension.

### Activation of ^166^Ho microparticles suspension

For each tumor, a volume of 800 µl of the ^165^Ho suspension was withdrawn from the initial vial and filled in a thermoplastic PolyEtherEtherKetone (PEEK) capsule suitable for the activation process. The ^166^Ho, T_1/2 =_ 26,8h, was then produced within the suspension of microparticles with a neutron activator (Advanced Accelerator Applications: AAA) coupled to a 70MeV cyclotron (IBA, C70 of GIP Arronax facility). The principle of the activation consisted of the production of a neutron flux generated by the interaction of a highly intense high energy proton beam (350 µA - 70 MeV) on a metallic target chosen to produce a high neutron flux. The neutrons then interact with the suspension of microparticles and through neutron capture, a small part of the stable atoms of ^165^Ho is transformed into radioactive ^166^Ho. In nuclear physics, this neutron capture reaction is written as ^165^Ho (n,γ) ^166^Ho since, when capturing an incident neutron, the nucleus also emits a gamma ray to compensate the excessive energy provided by the neutron when returning to his fundamental energy state. At the end of the production, the vials are automatically unloaded from the irradiation bunker and the activity of each vial is measured using a dose calibrator (accuracy 5%). In this study, samples were activated with irradiation time and current intensity allowing to deliver at time of injection increased local therapeutic absorbed dose as compared to the standard EBRT target level (60 Gy in the whole tumor volume). Here, the samples were produced in order to obtain specific activities at the time of injection of 2 to 3 MBq/mg of microparticles. This level of specific activity was then used as an input parameter for the treatment planning system (TPS) i.e., the developed algorithm used to determine the number of injections and their location to satisfy the dosimetric requirements. After activation, the vials were placed in a lead pot and sent to the preclinical center the day before treatment. On the day of treatment, the content of each vial was mixed to get a homogeneous solution in a dedicated injector device, allowing safe operation for the users. Finally, the product was ready for intratumoral injection.

### Preclinical injection system

The prototype injection system of Ho suspension was designed by EVEON to inject a precise volume of Ho suspension as previously described (17). With the use of this preclinical injection system, it was possible to cover a large volume of the tumor in large animals with Ho suspension. Tumor sizes should be between 1 and 3 cm in diameter, with larger brain tumors considered as several tumors of 1 to 3 cm. The main parts of this system were described in a previous study (63). In summary, it includes: 1) Injector; 2) Injector control software; 3) Injection needle; 4) Needle housing which has a multiple roles, including operating and guiding the needle at the right location and position of each unit injection and in the radioprotection when withdrawing the needle contaminated with radioactive suspension from the tumor; 5) prefilled capsules of Ho suspension; 6) Lead container to hold the radioactive suspension capsule and provide gamma shielding; 7) Disposable fluidic cassette. The injection technique was precisely explained in a previous study on intratumoral injection of ^165^Ho (17). Briefly, the stereotactic frame was used to manually deploy the injection needle at a given position in the tumor. The Ho suspension is then automatically injected by the execution of a command through the injector control software. This allows the suspension to be injected with predefined unit volume and flowrate. This operation was then repeated for all injections, without ever removing the injection needle from the tumor.

### Treatment planning system

As detailed in Brown et al. (22), a bespoke TPS was created to generate proposed mBT treatment scheme. The algorithm attempted to deliver at least 95% of the target absorbed dose, set at 100 Gy, to at least 95% of the gross tumor volume (GTV) using the fewest injections possible. Injection volumes were allowed to vary between 5 and 8 μl and are referred to as Unit of Treatments (UoTs).

A pre-operative CT scan (140 keV), typically acquired 30 minutes before the injection of ^166^Ho suspension, was used for the treatment planning (TP). Performing the pre-operative CT scan as close to the time of intervention as possible meant that the planning was performed with the most up-to-date information regarding the tumor’s size, shape and position.

The CT acquisition was acquired with product contrast to aid the differentiation between the tumor and healthy tissue.

### Dosimetry

Quantification was performed by first establishing a calibration curve from the CT scan in order to calculate Ho microparticle distribution from the signal produced by the CT. If, in the future, this treatment was performed at a larger scale, the calibration curve should be established for each CT scanner with a standardized protocol before the start of the therapeutic process as a means to ensure that the absorbed dose distribution center can accurately evaluated.

Determination of the distribution of the injected Ho from the post-operative CT scan led to the resulting absorbed dose distribution. This was achieved by assuming no biological clearance of Ho particles (radioactive decay by physical half-life of ^166^Ho only) and using GATE (23), a radiation transport simulation platform based on the Monte Carlo code Geant4 (24).

### Immunosuppressive therapy

The immunosuppression procedure and surgical procedure of U87 cells implantation was carried out as previously described (17, 19). Briefly, cyclosporine solution (Neoral^®^ 100 mg/ml) was administered orally, twice a day (25 mg/kg). The cyclosporine treatment continued each day until the last day of the experiment. Generally, the blood level of cyclosporine was maintained above 1000 ng/ml so as to increase the probability of tumor development. In this regard, blood sampling was performed at least once a week to monitor the cyclosporine serum concentration.

### Tumor cell transplantation and intratumoral injection of ^166^Ho microparticles

Twelve immunosuppressed Yucatan minipigs were anesthetized and each had the tumor cells injected into the brain in the corpus striatum area in the right hemisphere at D0. One single tumor developed unilaterally in the right side. Fourteen days after tumor cell transplantation, the minipigs were divided into three groups: Group 1 (n=6) received injections of ^166^Ho suspension (radioactive), group 2 (n=3) received injections of stable ^165^Ho suspension (non-radioactive) and group 3 as the control group (n=3), did not receive any injections. One of the 6 minipigs in group 1 did receive ^166^Ho suspension (radioactive) injection but due to a positioning issue, the suspension was placed outside the tumor. This minipig was excluded from the group 1 and named “Positive fail pig”. This case was named as such because, although the treatment was unfortunately unsuccessful, it highlighted some interestingly positive results, which will be discussed later.

A few minutes before starting the injections in group 1, the lead pot containing the Ho suspension housed in thermoplastic pharmaceutical grade capsules was transferred to the operation room for mounting into the injection system. The pigs’ heads were fixed in the stereotactic system and the injection needle was installed on the stereotactic manipulator. According to the tumor position, determined by the pre-operative CT images acquired just before the Ho suspension injection, the needle was inserted into the brain through the holes that had been drilled at D0 for U87 cells implantation. Several UoTs were injected inside the tumor in different sites using the coordinates given by the TPS. The number of injections per tumor depended on the tumor size and shape. For all minipigs, the injection rate was set at 50 μl/min. The needle housing was positioned on the tray beside the animal to put the injection needle inside it immediately after withdrawing from the brain.

After treatment of each animal, all the equipment, surgeons and the operation room were checked by an advanced radiation detector to identify any potential radioactive contamination on the staff and in the room.

### CT imaging

To study the distribution of Ho inside the tumor and evaluate the development of tumor size over time, CT acquisitions were periodically performed. A CT scanner (GE BrightSpeed 16) was used at Voxcan to perform CT acquisitions. The imaging characteristics and CT acquisition protocol before and following the U87 cells implantation was described in previous studies (17, 19).

Pre- and post-operative CT imaging was performed on the day of Ho suspension injection (D14) to assess tumor positioning and verify the distribution and location of injected Ho, respectively. After the treatment, the evolution of injected Ho suspension and the therapeutic effect of this radiation therapy were studied in group 1 (radioactive) and 2 (non-radioactive) by performing the first post-injection CT scan, 5 days after the intervention and then every ten days. For all minipigs, a manual segmentation of the tumor was performed after each post-operative CT acquisition. In this fashion, the changes of tumor size could be studied over time.

### Follow-up

A standardized follow-up period was used for the 2 months after treatment. In this period, all the animals were monitored for clinical side effects on a daily basis.

Blood samples were taken at least once a week, to check renal and hepatic parameters and to evaluate the presence of any infection or inflammatory process. In this period, reasons for sacrificing the minipigs were severe ataxia, lateral recumbency, anorexia, head tilt and complete apathy. When these neurological symptoms were observed, the animal was euthanized by administration of 30 ml pentobarbital (Dolethal^®^) 200 mg/ml, intracardially.

### Histology and immunohistochemistry

Samples from brain tumor, heart, liver, lung and kidney were collected, formalin-fixed, and routinely processed into paraffin blocks. For each sample, a 4 μm section was stained with hematoxylin and eosin (H&E). For each brain sample, 7 sections were used for IHC staining, using antibodies at previously established dilutions: 10µg/ml anti-CD3 epsilon (CD3-12, BioRad) and 250µg/ml anti-Pax-5 (24/Pax5, BD Biosciences) for T and B lymphocytes, 5µg/ml anti-CD204 (SRA-E5, Abnova) and 5µg/ml anti-CD206 (122D2.08, Bio techne) for macrophages. Antigen retrieval was performed by heating at 90 °C for 40 minutes in citrate tampon pH 6 (ThermoScientific Dewax and HIER Buffer L, ThermoFisher, Runcorn, United Kingdom), followed by a cool off. Labeling was amplified using the ultraTek HRP (anti-polyvalent) Ready to use kit (ScyTek), revealed with Vector NovaRED Peroxidase (HRP) Substrate kit and counterstained with hematoxylin. According to standard IHC, negative and positive controls were used.

### Scoring and pattern identification at light microscopy

On the H&E stained section of the brain, the following parameters were scored: presence/absence of tumor, presence/absence of Ho crystals, tumor necrosis, lymphocytic inflammation and granulomatous inflammation. For tumor necrosis, the following semi-quantitative score was applied: (-) = absent, (+) = 1-30% of the tumor area, (++) = 31-60% of the tumor area, (+++) = 61-100% of the tumor area. Tumor lymphocytic and tumor granulomatous inflammations, as well as CD3, Pax5, CD204, and CD206 positive cells were evaluated semi-quantitatively as follows: (-) = absent, (+) = mild, (++) = moderate, (+++) = severe. All the histological and immunohistochemical parameters were scored blindly.

### Statistical analysis

Survival data were analyzed using a log-rank test (Mantel-Cox). Statistical analyses were conducted using this non-parametric test, which is used in preclinical and clinical trials to establish the efficacy of a new treatment in comparison with a control treatment. A value of p < 0.01 was taken to indicate statistical significance.

## Results

### Tumor growth following U87 implantation

**Figure 1:**
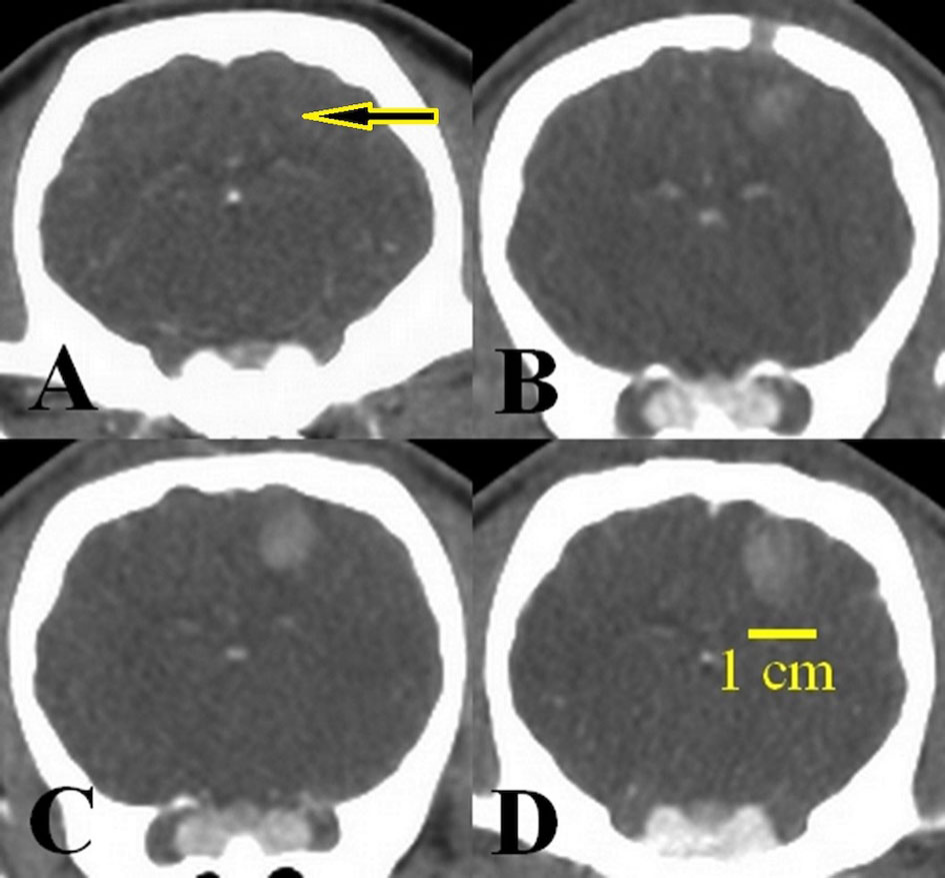
Tumor growth over 14 days post-implantation of the U87 cell line. **(A)** Cell implantation area (arrow) at day 0, **(B)** tumor size 4 mm, 7 days after implantation, **(C)** tumor size 7 mm, 10 days after implantation, **(D)** tumor size 13 mm, 14 days after implantation, just before injection of the Ho suspension.

In all 12 Yucatan minipigs, the post-implantation CT acquisitions leading up to D14 demonstrated tumor development at the injection site in corpus striatum of right hemisphere (**Figure 1**). Following the implantations, the whole-blood concentration of cyclosporine in all 12 animals was kept above 1000 ng/ml, which prevented tumor rejection or regression over the study period. From D0 to D14, no infections or neurologic symptoms were observed. The tumor diameter at the day of Ho injection (D14) varied between 1 and 2 cm.

### Intratumoral injection of Ho suspension

In groups 1 and 2, the number of injected UoTs varied between 9 and 20 depending on the tumor size and shape (**Table 1**). The time needed for the execution of a treatment (both ^166^Ho and ^165^Ho) for each pig was about 2 hours, including the pre-operative CT scan, TPS calculation and all pre-, per- and post-operation procedures. Also, the average time for injecting each UoT was about 1 minute. After intratumoral injection of the Ho suspension in groups 1 and 2, no backflow was observed in the holes regardless of the different volumes that were intratumorally injected (**Table 1**).

**Table 1:** Number and volume of unit of treatments (UoTs) injected in each animal.

Pig	Groups	Type of treatment	Number of	Volume of injected UoTs (µl)
			injected UoTs	
1	“Positive fail pig”	^166^Ho	9	3 x 5µl
				6 x 6µl
2	Group 1	^166^Ho	15	3 x 5µl
				4 x 6µl
				8 x 8µl
3		^166^Ho	20	8 x 5µl
				12 x 6µl
4		^166^Ho	13	13 x 5µl
5		^166^Ho	16	13 x 5µl
				3 x 6µl
6		^166^Ho	20	11 x 5µl
				9 x 6µl
7	Group 2	^165^Ho	11	9 x 5µl
				2 x 6µl
8		^165^Ho	20	16 x 5µl
				4 x 6µl
9		^165^Ho	20	3 x 5µl
				17 x 6µl
10	Control group	–	–	–
11		–	–	–
12		–	–	–

**Figure 2:**
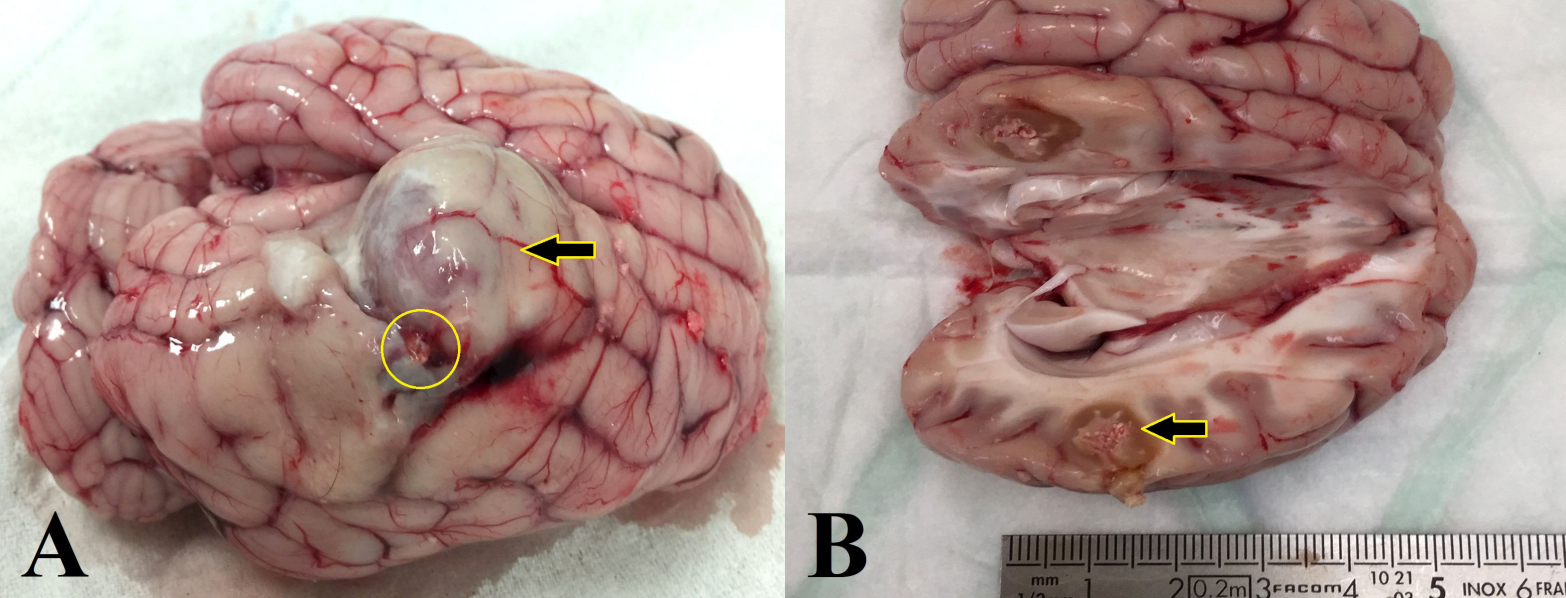
Macroscopic aspect of the tumor-bearing brain after the treatment with ^166^Ho suspension. **(A)** Ho injection outside of the GB tumor (yellow circle) for the pig 1, “positive fail pig”. Tumor growth continued on the opposite side of the ^166^Ho injection area (arrow). **(B)** Pig of group 1, ^166^Ho was injected entirely inside the tumor in the targeted location (arrow). No evidence of hemorrhage due to needle penetration.

After removing the tumor-bearing brain, the injected Ho was observed inside all tumors (**Figure 2B**), except in one, “Positive fail pig”, in which the injected liquid was found in the lateral part of the tumor. In this case, a large GB tumor was observed (**Figure 2A**). Visually, there was no evidence of hemorrhage due to the needle penetration (**Figure 2B**).

### Survival time

**Figure 3:**
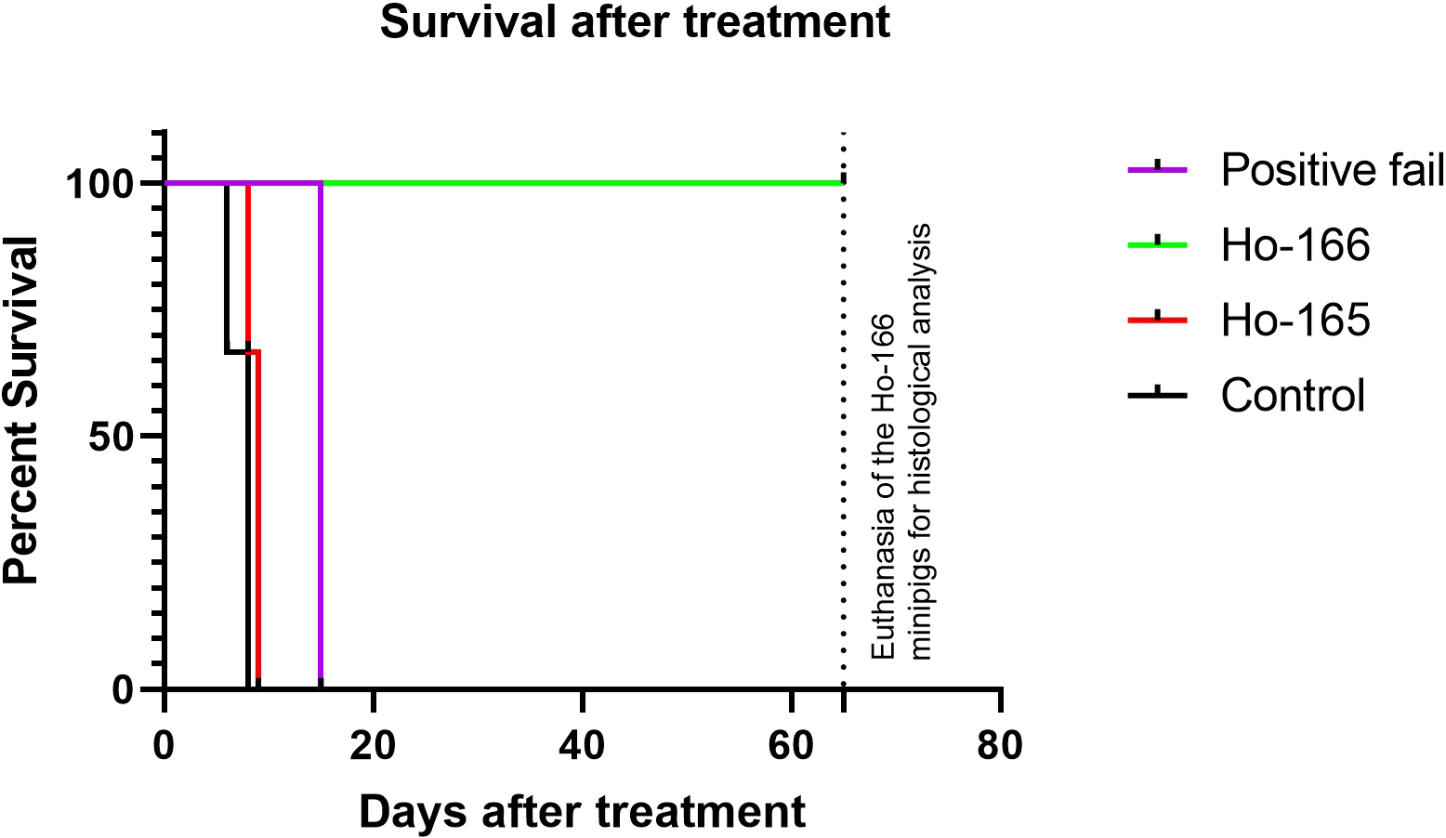
Kaplan-Meier survival curves of group 1 (^166^Ho), group 2 (^165^Ho), the control group and the “positive fail pig”.

The survival time was truncated 2 months after treatment in order to conduct histological analyses. The Kaplan-Meier survival curve (**Figure 3**) was the longest in group 1 treated with radioactive ^166^Ho. For group 2 and the control group, the survival curves were almost the same as well as the one obtained with the “positive fail pig”.

The survival period for all animals was calculated from the date of Ho injection at day 14 post-implantation (D14). In group 2 and the control group, all minipigs demonstrated severe neurologic signs between 6 and 9 days after D14 and consequently they were euthanized. In group 1, five minipigs survived until the end of the observation period, 2 months after D14. At the time of euthanasia, 5 out of 5 minipigs in group 1 were healthy, in a good general condition. The “positive fail pig” developed untreatable neurological symptoms, leading to euthanasia 15 days after the treatment. The survival times for all minipigs are presented in **Table 2**. According to statistical analysis, the survival rate of group 1 was significantly different from the two other groups (p < 0.01). There was no significant difference between group 2 and the control group (p=0.09).

**Table 2:** Survival time of each group including the one for the “positive fail pig”.

Group 1 (treated with ^166^Ho). n = 5 minipigs	Positive fail pig	Group 2 (injected with ^165^Ho). n = 3 minipigs	Control group
	n = 1 minipig		n = 3 minipigs
66	15	9	8
66		8	6
66		9	8
66		Mean: 8.6 days	Mean: 7.3 days
66			
Mean: 66			

### CT imaging

**Figure 4:**
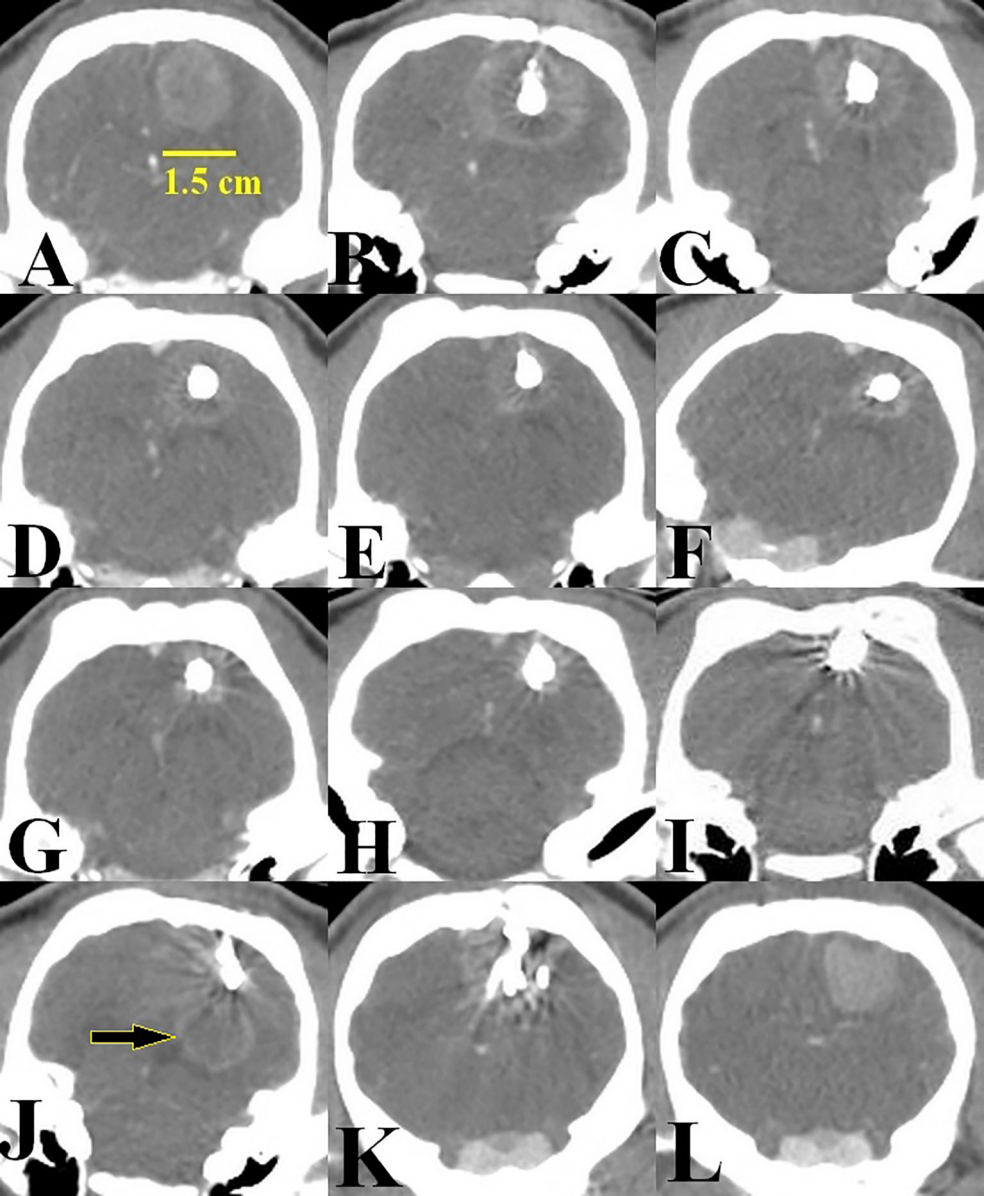
Representative example of the images acquired from D14 onwards. **(A)** D14, tumor size 1.5 cm just before Ho injection, **(B)** D14, presence of 166Ho just after intratumoral injection, group 1, **(C)** 5 days after intratumoral injection of 166Ho. Tumor size is regressing, **(D)** 15 days after intratumoral injection of ^166^Ho. Tumor size is regressing, **(E)** 25 days post-injection of ^166^Ho, **(F, G)** 45 and 55 days post-injection of ^166^Ho. A small volume of tumor is observable, **(H)** 65 days post-injection of ^166^Ho, just before animal euthanasia at the end of the observation period (D80), **(I)** Tumor condition after intratumoral injection of ^166^Ho, just before animal euthanasia. This image was acquired from the minipig in which the tumor was no longer observable from 45 days post-injection of ^166^Ho, **(J)** CT scan of the minipig “positive fail pig”. Tumor continued to growth (arrow), **(K)** CT scan of a minipig from group 2 (^165^Ho) acquired before euthanasia, **(L)** CT scan of a minipig from the control group acquired before euthanasia.

Several CT acquisitions were performed (**Figure 4**) before and after injections of the Ho suspension in groups 1 and 2. At D14, all the implanted tumors demonstrated an increased density in comparison with the surrounding cerebral parenchyma, with clear boundaries and a plurilobed form (**Figure 4A**). There was no hemorrhage after cell implantation. According to the post-operative CT scans in groups 1 and 2, substantial accumulation of Ho microparticles was seen at the injection sites (**Figures 4B, K**). This indicated that the Ho suspension had been successfully injected intratumorally in all but one minipig “positive fail pig”. In this animal, the majority of the ^166^Ho was injected outside the tumor (**Figure 4J**). CT images showed that the Ho suspension was localized and remained at the injection sites over 2 months post-injection, validating the assumption of no biological clearance stated in the Dosimetry section.

According to CT images acquired after D14, tumors regressed significantly in group 1 (**Figures 4C, D, E, F, G, H**) and were not even observable in 3 minipigs, 45 days after injections of Ho (**Table 3**, **Figure 4I**). Yet in groups 2 and the control group, tumor growth continued (**Figures 4K, L**).

**Table 3:** Tumor volumes (mm^3^) of the 12 minipigs.

Group	Pigs	Volume tumor (mm^3^)										
		D0	D7	D10	D14*	D19	D29	D40	D50	D60	D70	D80
“Positive fail pig”	1	0	218	419	871	2843	11124, EU.+	EU. D29	EU. D29	EU. D29	EU. D29	EU. D29
1	2	0	263	1064	2710	6787	3173	1726	2045	2259	2319	1596
	3	0	279	543	1789	4863	UO.^§^	UO.	UO.	UO.	UO.	UO.
	4	0	222	460	1108	2788	5724	4759	UO.	UO.	UO.	UO.
	5	0	170	325	1210	3099	4112	1208	613	UO.	UO.	UO.
	6	0	322	239	694	3229	7259	2083	958	905	806	800
2	7	0	181	262	579	2714	EU. D23	EU. D23	EU. D23	EU. D23	EU. D23	EU. D23
	8	0	390	572	2264	8010	EU. D22	EU. D22	EU. D22	EU. D22	EU. D22	EU. D22
	9	0	316	399	1191	5237	EU. D23	EU. D23	EU. D23	EU. D23	EU. D23	EU. D23
Control	10	0	481	707	787	2511	EU. D22	EU. D22	EU. D22	EU. D22	EU. D22	EU. D22
	11	0	157	342	1179	5419	EU. D20	EU. D20	EU. D20	EU. D20	EU. D20	EU. D20
	12	0	505	743	928	1694	EU. D22	EU. D22	EU. D22	EU. D22	EU. D22	EU. D22

*The day of Ho injection.

+Euthanized at:.

§Unobservable.

Volumes are based on several CT images acquired after U87 cell implantation. At D14, 166Ho and 165Ho were injected intratumorally in groups 1 and 2, respectively.

### Dosimetry

While performing the dosimetry evaluation, it was observed that the tumors were mostly under-irradiated compared to the initial absorbed dose target, i.e., 100 Gy to over 95 % of the tumor volume. This can be seen in **Table 4**, in which the percentage volume of the tumor receiving 60 Gy (the absorbed dose typically prescribed in EBRT) and 100 Gy (the absorbed dose target for this treatment) are shown.

**Table 4:** Tumor volume coverage for all the pigs treated with 166Ho for two typical values of absorbed dose, 60 and 100 Gy.

Groups	Pigs	Tumor volume coverage at 60Gy (V_60 Gy_) *	Tumor volume coverage at 100Gy (V_100 Gy_)	Status
“Positive fail pig”	1	55.50%	49%	Euthanized
Group 1	2	51.40%	44.30%	> 2 months survival
	3	43%	37.10%	> 2 months survival
	4	58.50%	50.20%	> 2 months survival
	5	99.30%	97.40%	> 2 months survival
	6	84.30%	79.10%	> 2 months survival

* 60Gy is the typical target value used for EBRT with a coverage of 98% of the tumor volume.

### Histology

**Figure 5:**
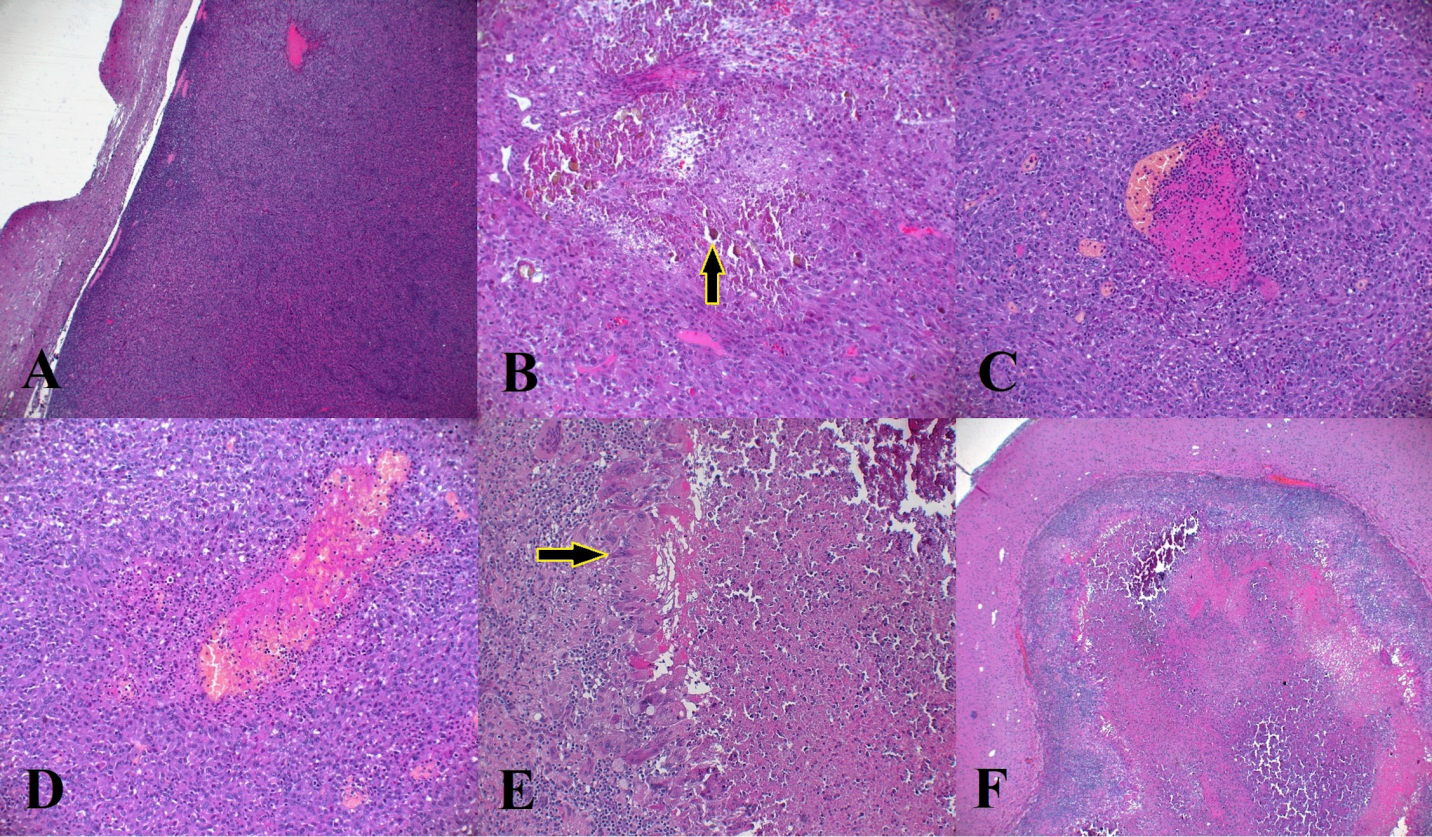
Microscopic findings of U87 tumor in the 3 groups. **(A)** Delimited tumor from the adjacent parenchyma. Tumor is highly cellular, **(B)** presence of Ho Crystals (arrow) located in necrotic areas within the tumor, **(C, D)** coagulative necrosis located around the blood vessels in control group, **(E)** Granulomatous inflammation in group 1 composed of macrophages and multinucleated giant cells, **(F)** severe necrosis in the tumors treated with ^166^Ho (group 1).

By H&E staining, tumor was detected in all the animals of group 2 and control group, and in the “positive fail pig”. In group 1, a proper mass was not detected: necrosis and inflammation were so severe (**Figures 5D, E**) that residual tumoral cells, if present, were probably dispersed and they were not easily recognizable. Ho crystals were observed in necrotic areas within the tumor in groups 1 and 2 (5/5 group 1, 3/3 group 2) (**Figure 5B**). Necrosis was detected in almost all the animals (5/5 group 1, 3/3 group 2, 2/3 control group and the “positive fail pig”). In group 2 and the control group, necrosis was less than 60 % of the tumoral surface. There was mainly a coagulative necrosis centered on blood vessels (**Figures 5C, F**), which were obliterated by thrombi. In group 1, necrosis was severe, replacing the tumor, which was no longer identifiable in 5/5 animals. (**Figure 5E**). There was a liquefactive necrosis surrounded by a granulomatous inflammation. Granulomatous inflammation (**Figure 5D**) was absent in control group (0/3 animals); it was mild in group 2 (3/3 animals), and moderate to severe in group 1 (5/5 animals). Lymphocytic inflammation was present in all the groups, being slightly more severe in group 1 (**Table 5**).

**Table 5:** The findings observed on H&E slides in the brain of the animals in groups 1, 2, control group and positive fail pig.

Groups	Pigs	Tumor	Holmium	Necrosis	Lymphocytic Inflammation	Granulomatous Inflammation
“Positive fail pig”	1	Present	Absent	+++	++	–
Group 1	2	Not clearly visible	Present	+++	+++	++
	3	Not clearly visible	Present	+++	++	+++
	4	Not clearly visible	Present	+++	+++	++
	5	Not clearly visible	Present	+++	+++	+++
	6	Not clearly visible	Present	+++	+++	++
Group 2	7	Present	Present	++	++	+
	8	Present	Present	+	++	+
	9	Present	Present	++	+	+
Control group	10	Present	Absent	+	++	–
	11	Present	Absent	–	++	–
	12	Present	Absent	+	+	–

### Immunohistochemistry

In all brain samples, most of the tumor-associated lymphocytes inside the tumor were CD3+ T-lymphocytes, with only a few dispersed Pax-5+ B lymphocytes.

**Figure 6:**
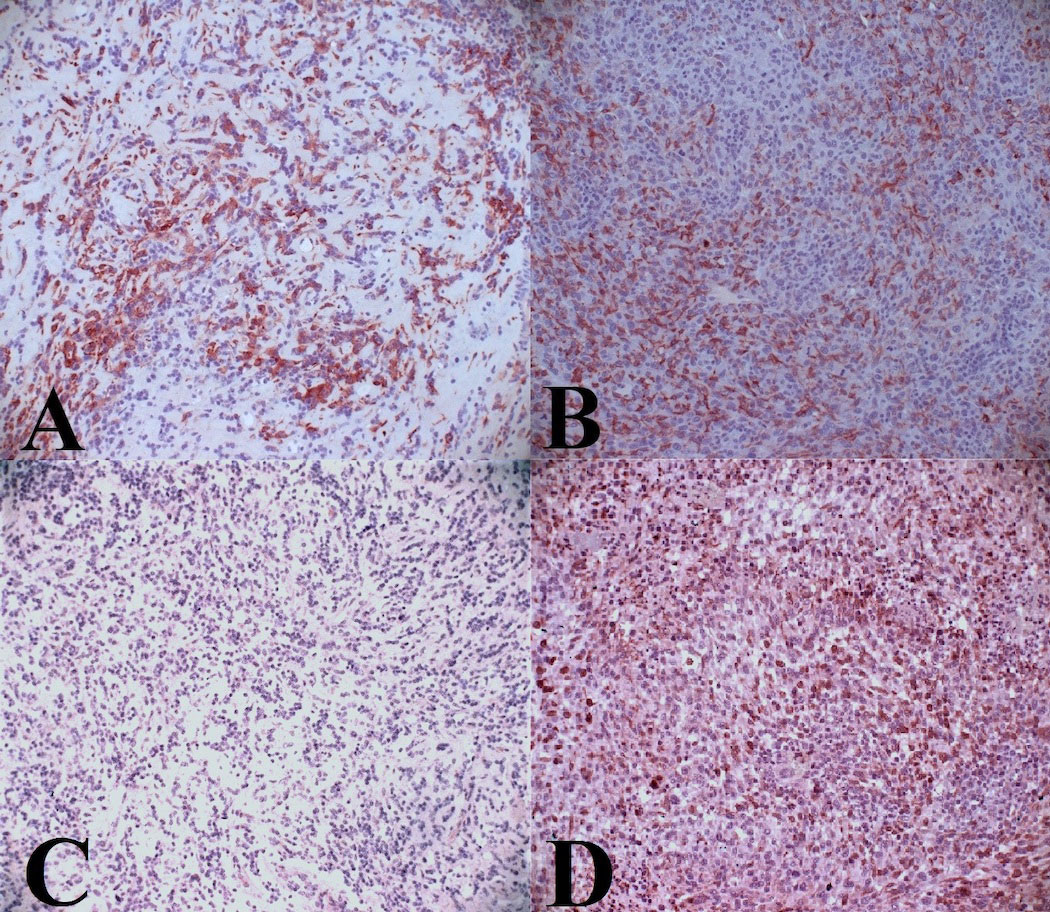
Representative of CD204 and CD206 marker. **(A)** Intratumoral infiltration of CD204. Moderate positive labeling in group 1, **(B)** intratumoral infiltration of CD204. Moderate positive labeling in control group, **(C)** Absence of intratumoral infiltration of CD206 in group 1, **(D)** Intratumoral infiltration of CD206. moderate to severe positive labeling in group 2.

A moderate amount of CD204+ macrophages were observed, without any relevant difference among all groups (**Figure 6** and **Table 6**). CD206^+^ macrophages were increased within the tumor in group 2 and the control group, while they were almost absent in group 1 (**Table 6** and **Figure 6**).

**Table 6:** Evaluation of macrophage and dendritic cells infiltration in the brain tumor.

Groups	Pigs	Intratumoral infiltrate	
		CD204	CD206
“Positive fail pig”	1	++	+++
Group 1	2	++	–
	3	+++	+
	4	++	–
	5	++	+
	6	+++	–
Group 2	7	++	+++
	8	++	++
	9	++	++
Control group	10	++	+++
	11	++	++
	12	++	+++

## Discussion

The GB infiltration is a significant problem that makes the cure of this tumor impossible with current therapeutic methods (25, 26). In this study, the therapeutic efficacy of ^166^Ho suspension was evaluated after its intratumoral injection in a GB minipig model. In mBT, retention of the radioactive suspension in the injection area over time is very important to avoid its leakage outside the tumor. As can be seen in **Figures 2**, **4**, the Ho suspension was well circumscribed inside the tumor and following scans demonstrated good retention up to 2 months post-injection. This is an absolutely essential specificity to prevent collateral damages to healthy tissues of the brain. Furthermore, it allows for accurate dosimetry.

Our trial showed that the Yucatan induced GB can be curatively treated with ^166^Ho mBT. A positive response to treatment was obtained in 5 out of 5 treated animals (group 1), based on CT scan imaging and *post-mortem* histology. Moreover, all the 5 minipigs were in good general condition until euthanasia which confirms the absence of clinical side effects of mBT with ^166^Ho suspension. It is in accordance with some sparse previously published data on ^166^Ho efficacy, as in the orthotopic mouse model of renal cancer (27).

Our histological study showed severe and widespread necrosis within tumoral lesion, almost avoiding the detection of tumoral cells. There was a liquefactive necrosis, indicating massive cell death, such as is caused by radical oxygen species produced by ionization therapy (28, 29). In mice model, Lee et al. (30) showed radionecrosis of hepatoma, transplanted subcutaneously in the nude mouse, after an intratumoral injection of ^166^Ho. These results confirm the efficacy of ^166^Ho mBT in tumor cells destruction. The necrosis was surrounded by a moderate to severe granulomatous inflammation, suggesting a foreign body reaction towards ^166^Ho microparticles, severely amplified by an immune response to tumor cell antigens liberated by ^166^Ho-induced necrosis (**Table 5**).

On the other hand, the “positive fail pig” was euthanized 15 days after ^166^Ho injection because of severe neurological symptoms due to the tumor growth because of the injection of radioactive suspension by mistake in the lateral part of the tumor. In this case, the tumor volume at the day of euthanasia was approximately 12 times greater than at D14. With this “positive fail pig”, post-CT imaging showed that the tumor grew on the opposite side of the tumor relative to the injected ^166^Ho. The results of this minipig could almost be considered as a positive, confirming the efficacy of the ^166^Ho when correctly injected. Further explanation as for which the radioactive suspension needs to be inside the tumor is given below.

As in the rodent model, Huh et al. have reported that ^166^Ho chitosan complex can be used for the treatment of malignant glioma in mice model when injected at the appropriate absorbed dose and area (16).

In dosimetry analysis, as shown in **Table 4**, one slightly surprising result was that the tumors were under-irradiated compared to their target absorbed dose distributions. We believe the lower-than-intended absorbed dose distribution is due to the novel injection method and some physical limitations encountered for the needle positioning. In standard brachytherapy (BT), many needles traverse healthy brain tissue and are inserted into the target volume, with radioactive sources placed along these paths (either temporarily or permanently). The novelty of the proposed mBT treatment is that with a single needle crossing the healthy tissue, a large portion of the tumor can be accessed. Table 4 indicates that this prototype method requires slight enhancement to be able to irradiate the whole tumor volume.

However, modification of the injection method may not be necessary given the positive results exhibited by group 1 (seen in **Figures 3**, **4I** and **Tables 2**, **3**). Further, in group 1, there was no noticeable difference between the outcome of the pigs receiving the highest (V_100 Gy_ = 97.4%) and the lowest (V_100 Gy_ = 37.1%) irradiation coverage (**Tables 3**, **4**). This evidence indicates that, for mBT, moderate irradiation of the tumor is sufficient for tumor destruction. We believe that it might be due to an additional therapeutic effect on top of the cytotoxicity delivered by ^166^Ho ionizing radiation. We hypothesize that this effect might result from the presence of the radioactive Ho microparticles within the tumor that would participate in an additional immune activation, as discussed below.

In the immunohistochemistry analysis, we evaluated lymphocytic and macrophages infiltration to characterize the tumor immune microenvironment (TIME) as it plays a key role in the effectiveness of radiation therapy.

Lymphocytic infiltration, composed by T lymphocytes, was present in all the cases, without any significant difference among groups. We could assess the presence of neither cytotoxic T lymphocytes (CTLs) nor natural killers (NK), since no available antibodies work on paraffin embedded pig tissues. The severity of lymphocytic infiltration in group 1 was greater (not significantly) than in other groups (**Table 5**) which might be due to the effect of radiation induced tumor necrosis. It is known that localized irradiation of the tumor site can modify the microenvironments generating inflammatory cytokines, thereby increasing trafficking and retention of T lymphocytes within the tumor. In contrast, T-cells secrete cytokines affecting the activation and differentiation of the immune cells. They can also directly destroy cancer cells (31). This could improve the therapeutic efficacy of RT inside the tumor. Jarosz-Beij et al. have observed that a single absorbed dose of 10 and 15 Gy (High-dose rate iridium-192) in BT of mouse melanoma can show slight differences T lymphocytes infiltration compared to the control group. In contrast, the NK cell infiltration was increased twice in irradiated tumor (15 Gy) when compared to the control group (32). In this study, based on the observed differences between group 1 and the other two groups about lymphocytic infiltration, we can speculate that the observed necrosis in ^166^Ho treated animals might depend on a cytotoxic lymphocytic activity.

In addition to lymphocytes, the presence of TAMs inside the tumor was evaluated, using macrophages markers such as anti-CD204, and anti-CD206, extensively used in literature. (33, 34). The expression level of CD204 showed a large number of macrophages within the tumor of all the animals, without any differences among groups (**Table 6**). One the other hand, in many studies, CD206 was used as the standard biomarker to identify the M2-like phenotype of TAMs in glioma tissue (35–37). M2-like TAMs are immunosuppressive and promote tumor progression. They secrete anti-inflammatory cytokines such as IL-10, IL-13, and IL-4 and express abundant arginase-1, scavenger receptors and mannose receptor (34, 38). As can be seen in **Table 6**, it seems that the pro-tumoral M2-like macrophages were reduced or completely eliminated in group 1 following the mBT by ^166^Ho microparticles. Jarosz-Biej et al. showed that the area infiltrated by M2 macrophages decreased more than 3.5 times in the tumor following the BT of melanoma in mice model (32).

All these observations support a synergetic effect of the cytotoxicity due to ionizing radiation and an immune response coming from the presence of the radioactive ^166^Ho microparticles inside the tumor. The “positive fail pig” was also a good (albeit unexpected) comparison group as the ionizing radiation coverage was similar as other pigs of group 1, while the therapeutic effect was not present as the radioactive particles where not located inside the tumor mass.

Another hypothesis about the additional therapeutic effect following mBT is the radiation-induced bystander effect (RIBE). The radiological effect is transmitted from irradiated cells to near unirradiated cells, conducting to biological changes such as chromosomal instability, apoptosis, micronucleation and reduced clonogenic efficiency in the recipient tumoral cells (39, 40). This communicative effect is mediated by signaling through gap junctions and also through networks involving some tumor microenvironment cells, such as macrophages. Furthermore, another intercellular effect, called the Cohort effect, is defined as the interaction between irradiated cells inside an irradiated zone where the high-dose irradiated tumor cells might affect the low-dose irradiated tumor cells (41). This effect is usually limited to an area of millimeters (42). Studies are still ongoing to better understand the role of these effects on the tumor treatment following the RT.

In conclusion, through this study on the therapeutic effects of ^166^Ho microparticles injections in a GB tumor model in minipigs, the excellent efficacy of the tumor control has been demonstrated. ^166^Ho microparticles can be used to treat GB induced by implantation of U87 cells line which is considered as one of the most radioresistant cell lines (19–21). Regarding these encouraging results, we conclude that intratumoral treatment with ^166^Ho microparticles could be a good option for the treatment of GB as well as other solid tumors. The benefits of this method are largely due to the intensive irradiation of the tumor with the high-energy beta particles as well as a combined immune activation coming from the presence of the radioactive microparticles inside the tumors, thereby inducing severe central necrosis of the tumor without direct impairment or side effects to the surrounding tissues. Another advantage of this novel mBT in comparison with other treatment modalities is the single session approach and its minimally invasive nature, without the severe secondary effects that are often encountered with chemotherapy and other radiotherapy methods. In particular, the flexibility observed in term of dosimetry is totally opposite to the requirements existing in EBRT demonstrating here an innovating new therapy modality which open the way to better treatment efficacy. Overall, further studies on the long-term efficacy and toxicity of these microparticles are needed. Also, more investigations are required to more precisely identify the type of TAMs following ^166^Ho mBT in order to unravel the immunological mechanism associated with this therapeutic method. Furthermore, association with a systemic administration of the gold standard Temozolomide (TMZ) chemotherapy drug could be investigated to improve therapeutic efficacy.

In future, this microbrachytherapy method would be translated into a clinical phase 1 trial including a small number of patients in order to assess tolerance and efficacy of the intra-tumoral suspension injection in GB brain tumors. Patient population relevant for such study would be relapsed patients after first line of treatment. IRM and SPECT-CT imaging modalities would be appropriate tools for pre-selection process and patient’s follow-up after treatment in order to monitor microparticles distribution and dosimetry (43).

## Data availability statement

The original contributions presented in the study are included in the article/supplementary material, further inquiries can be directed to the corresponding author/s.

## Ethics statement

This research is in accordance with the French Ministry of Scientific Research (2015052012034148 v1) and approved by the VetAgro Sup Ethical Committee (1522 V2).

## Author contributions

FP, IZ, MB, LM and MM designed the research project. FP, TR and CC supervised CQ’s Ph.D student. CC, TB and MK performed minipig neurosurgery. SB, CB-R and EP-M performed immunohistochemistry staining, and scoring and data analysis. MK, RB drafted the manuscript and all other authors critically reviewed the manuscript and approved the final version submitted. All authors contributed to the article and approved the submitted version.

## Funding

This work was supported by the BPIfrance through the TheraneaM project [ISI 2013–2018] coordinated by Advanced Accelerator Applications.

## Acknowledgments

We would also like to thank Meriadeg Guillamet, Jean Michel Buhour, Gilles Bouvet for their role in the development of this project, especially the mechanical part associated to the neutron activator. We gratefully acknowledge the Eveon company for their contribution in the development of the injector system.

Frédérique Ponce and the whole team dedicate this article to our collaborator and friend Dr Claude Carozzo, who left too brutally and too quickly.

## Conflict of interest

The authors declare that the research was conducted in the absence of any commercial or financial relationships that could be construed as a potential conflict of interest.

## Publisher’s note

All claims expressed in this article are solely those of the authors and do not necessarily represent those of their affiliated organizations, or those of the publisher, the editors and the reviewers. Any product that may be evaluated in this article, or claim that may be made by its manufacturer, is not guaranteed or endorsed by the publisher.
